# Cortical modulation of auditory processing in the midbrain

**DOI:** 10.3389/fncir.2012.00114

**Published:** 2013-01-03

**Authors:** Victoria M. Bajo, Andrew J. King

**Affiliations:** Department of Physiology, Anatomy and Genetics, University of OxfordOxford, UK

**Keywords:** auditory cortex, inferior colliculus, corticofugal, descending projection, plasticity, sound localization

## Abstract

In addition to their ascending pathways that originate at the receptor cells, all sensory systems are characterized by extensive descending projections. Although the size of these connections often outweighs those that carry information in the ascending auditory pathway, we still have a relatively poor understanding of the role they play in sensory processing. In the auditory system one of the main corticofugal projections links layer V pyramidal neurons with the inferior colliculus (IC) in the midbrain. All auditory cortical fields contribute to this projection, with the primary areas providing the largest outputs to the IC. In addition to medium and large pyramidal cells in layer V, a variety of cell types in layer VI make a small contribution to the ipsilateral corticocollicular projection. Cortical neurons innervate the three IC subdivisions bilaterally, although the contralateral projection is relatively small. The dorsal and lateral cortices of the IC are the principal targets of corticocollicular axons, but input to the central nucleus has also been described in some studies and is distinctive in its laminar topographic organization. Focal electrical stimulation and inactivation studies have shown that the auditory cortex can modify almost every aspect of the response properties of IC neurons, including their sensitivity to sound frequency, intensity, and location. Along with other descending pathways in the auditory system, the corticocollicular projection appears to continually modulate the processing of acoustical signals at subcortical levels. In particular, there is growing evidence that these circuits play a critical role in the plasticity of neural processing that underlies the effects of learning and experience on auditory perception by enabling changes in cortical response properties to spread to subcortical nuclei.

The traditional view of the central auditory pathway begins with the auditory nerve fibers entering and bifurcating in the cochlear nuclei from where information is transmitted successively via other centers in the brainstem, midbrain, and thalamus to the auditory cortex, where further processing gives rise to our perception of the auditory world. According to this hierarchical view of the ascending pathways, the inferior colliculus (IC) in the midbrain is usually regarded as an obligatory relay for the transmission of auditory signals (e.g., Aitkin and Phillips, 1984). When a retrograde tracer injection is placed in the IC, however, more cells are labeled in the auditory cortex than in brainstem centers such as the cochlear nuclei or superior olivary complex (Figure 1A). Consequently, processing in the IC must be influenced by cortical outputs. In fact, descending projections connect almost all levels of the auditory pathway, indicating that a bidirectional flow of information takes place.

**Figure 1:**
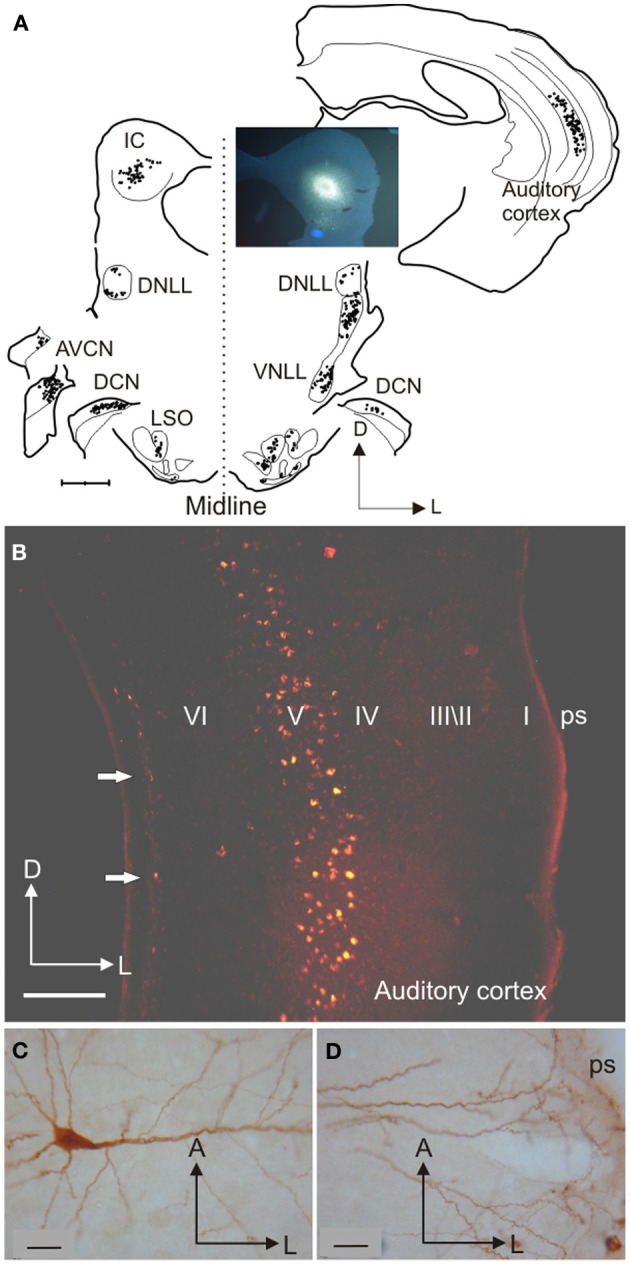
**Inputs from different auditory centers converge in the IC. (A)** A small fluorogold tracer injection in the ventromedial part of the IC central nucleus of the gerbil produces retrograde labeling of neurons in the MSO, periolivary nuclei, VNLL, and A1 on the same side, in the cochlear nuclei and IC on the opposite side, and in the LSO, DCN, and DNLL on both sides. **(B)** Retrogradely labeled cells in the cortex are found mainly in layer V after a rhodamine tracer injection in the IC. **(C)** Large labeled pyramidal cell with the soma located in cortical layer V and **(D)** a tufted dendritic tree ending in layer I. Calibration bars: 1 mm **(A)**, 0.2 mm **(B)**, and 25 μm **(C,D)**. Modified with permission from Bajo and Moore (2005).

Even though descending pathways have been characterized in numerous species [reviewed in Winer (2003)], the function of different nuclei still tends to be thought of in terms of the serial processing of auditory signals. Thus, when a particular property, such as contrast gain control (Rabinowitz et al., 2011) or novelty detection (Ulanovsky et al., 2003), is identified in cortical cells, one immediate question is whether that property is genuinely cortical in origin or inherited from the auditory thalamus or midbrain. This issue is, of course, not limited to the auditory system, as all sensory pathways comprise a series of subcortical and cortical centers. But the auditory system stands out in having so many subcortical processing stations, and therefore presents a particular challenge for indentifying how ascending and descending signals interact to determine the response properties of the neurons found there.

In this review, we first discuss the anatomical organization of the descending projections from the auditory cortex to the IC, then look at how cortical inputs influence the response properties of IC neurons, and finally consider what the functions of corticocollicular modulation might be. Rather than viewing it as an independent set of ascending and descending connections, we suggest that it is more appropriate to consider the auditory system as a series of dynamic loops in which neural coding in the IC and other subcortical nuclei is continually updated by changes in activity at higher levels as much as by the signals received from the brainstem.

## Cortical cells that project to the inferior colliculus

Although studies demonstrating that lesions in the temporal lobe of the cerebral cortex result in axon degeneration in the midbrain date back more than 50 years, the first evidence using an anatomical tracing method for a descending projection from the auditory cortex to the IC was provided by Beyerl (1978). After making injections of horseradish peroxidase (HRP) in the central nucleus of the IC (CNIC) in rats, he found that layer V pyramidal cells were retrogradely labeled in the ipsilateral auditory cortex. Beyerl (1978) also reported that small HRP injections resulted in restricted patches of labeled neurons in the cortex, raising the possibility that this projection might be topographically organized in much the same way as the ascending projections to the IC from auditory brainstem structures. No labeling was reported, however, in other cortical layers or in the contralateral cortex, probably due to the short survival times (24–48 h) and the limitations of the tracer used.

**Figure 2:**
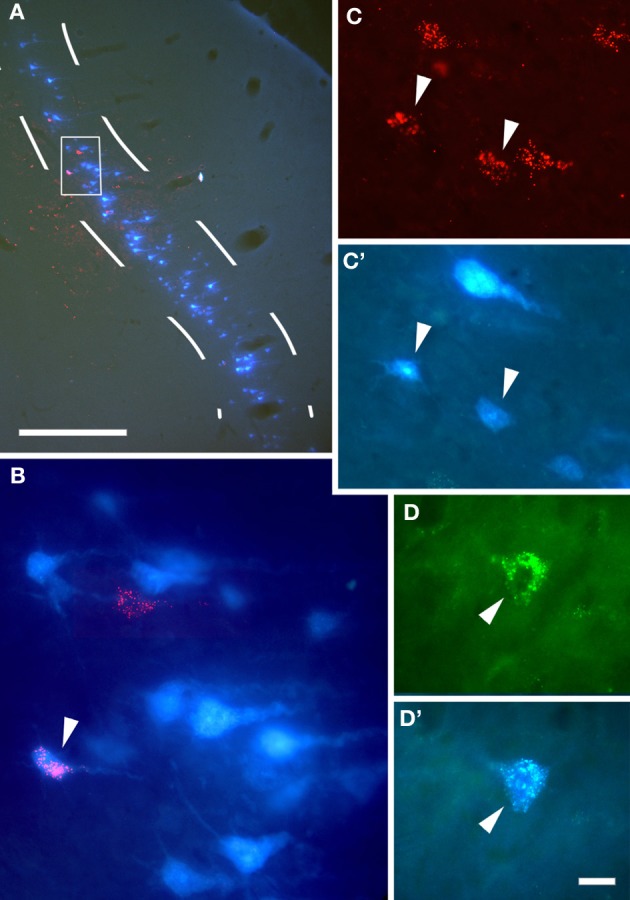
**Anatomical tracing experiments in guinea pigs show that cortical cells project to the IC bilaterally. (A)** Coronal section at the level of the right auditory cortex showing the overlay of cells labeled retrogradely by injections of Fast Blue in the right IC and rhodamine microbeads in the left IC, with a higher magnification of the area enclosed by the white box in **(B)**. **(C,C')** Double-labeled cortical cells (arrowheads) using the same tracer combination. **(D,D')** A double-labeled cortical cell following an injection of Fast Blue in the ipsilateral IC and fluorescein microbeads in the contralateral IC. Arrowheads indicate double labeled cells. Calibration bars: 0.5 mm **(A)** and 20 μm **(B–D)**. Modified with permission from Coomes et al. (2005).

**Figure 3:**
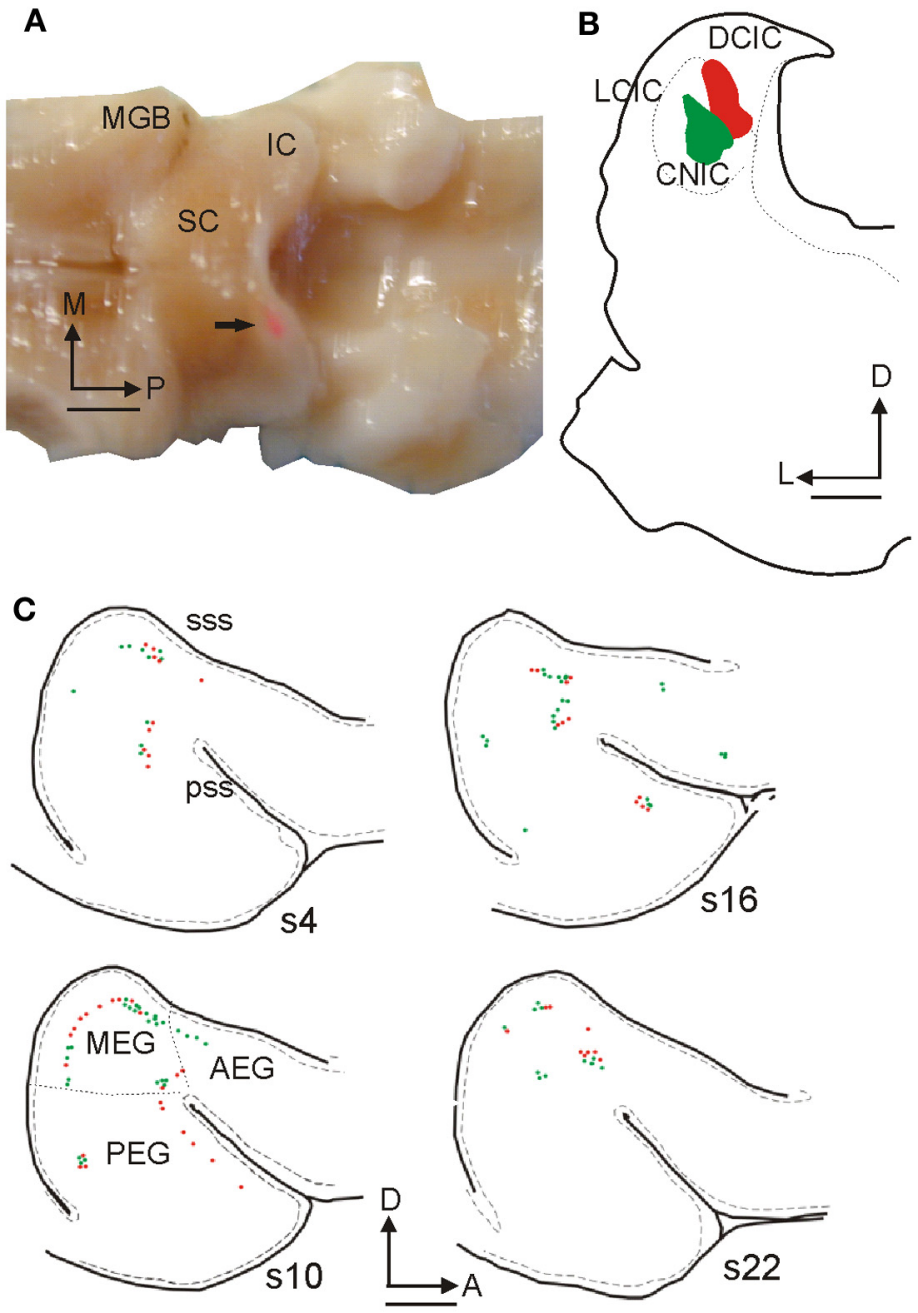
**Retrogradely labeled cells in ferret auditory cortex after fluorescent microbead injections in the IC. (A)** Dorsal view of the ferret brain where both the cerebral cortices and the cerebellum were removed to visualize the thalamus and midbrain. A rhodamine microbead injection site can be seen in the left IC (arrow). **(B)** Coronal section at the level of the IC from this animal illustrating rhodamine and fluorescein microbead injection sites. **(C)** Drawings of tangential 50 μm sections spaced 300 μm apart from lateral to medial (s22 is the most medial) at the level of the left ectosylvian gyrus where the auditory cortex is located, showing green and red retrogradely labeled cells. Calibration bars: 2 mm **(A,C)** and 1 mm **(B)**. Based on Bajo et al. (2007).

In the following decades, corticocollicular projections were described in squirrel monkeys (Winer et al., 2002), cats (Kelly and Wong, 1981; Winer and Prieto, 2001), ferrets (Bajo et al., 2007), guinea pigs (Strutz, 1987; Coomes et al., 2005), rats (Druga and Syka, 1984; Games and Winer, 1988; Herbert et al., 1991), gerbils (Bajo and Moore, 2005), and even in Madagascan tenrecs (Künzle, 1995). The use of modern retrograde tracers has confirmed that projection neurons that target the IC are found in layer V of the auditory cortex layer (Figures 1A,B and 2A) and revealed much about the morphology of these neurons (Figures 1–3).

Figure 1C shows a typical example of a large cortiocollicular pyramidal cell with a triangular cell body located in layer V. This neuron has 3–6 primary basal dendrites oriented parallel to the cortical layers, which branch to form a dendritic tree located mainly in the same layer, and a thick apical dendrite running perpendicular to the pial surface up to layer II and beyond, giving-off second-order dendrites along its length. When the IC cortices are included in the injection site in gerbils, the apical dendrites divide into 2–3 thinner dendrites, creating tufted apical endings in layer I (Figure 1D), suggesting that two different subpopulations of layer V pyramidal neuron, tufted and untufted, contribute to this projection (Bajo and Moore, 2005). However, these subpopulations have not been observed routinely in other species, such as ferrets (Bajo et al., 2007), possibly due to the incomplete filling of the distal dendritic arborization.

In another approach to examining the morphology of corticocollicular neurons, Games and Winer (1988) compared retrogradely labeled neurons in layer V with Golgi impregnated cells in rat cortex, and concluded that the IC projection neurons correspond to the medium- and large-sized pyramidal cells identified in Golgi-Cox preparations. Pyramidal cells in layer V are glutamatergic (Kaneko et al., 1987) and many of the corticocollicular projection neurons can be labeled using a specific antibody against the non-phosphorylated form of the neurofilament H protein SMI_32_ (Bajo et al., 2010a), although it is not known if every large neuron in layer V that projects to the IC is SMI_32_ positive or vice versa.

Layer V neurons are not the only cortical cells involved in the projection (Künzle, 1995; Doucet et al., 2003; Bajo and Moore, 2005; Schofield, 2009). When large tracer injections are placed in the IC, layer VI cells can also be labeled in the auditory cortex ipsilateral to the injection site (Figure 1B, arrows). Those labeled neurons located close to the border of the layer with the white matter (wm) are smaller than layer V projection neurons, and are not always pyramidal with some having an elongated soma often orientated parallel to the layers (Schofield, 2009). Although layer VI corticocollicular neurons are much less numerous than those found in layer V, they comprise about 10% of the population projecting to the ipsilateral IC and have a similar distribution across different cortical fields independent of the location of the injection site in the IC, except that very few are labeled by injections in the CNIC (Schofield, 2009).

The influence that layer V pyramidal cells exert on IC neurons may be very different from that of layer VI cells. Cortical layer V is both necessary and sufficient to produce synchronous cortical activity (Silva et al., 1991) and morphological evidence suggests that layer V projection neurons might correspond to the intrinsic bursting (IB) cells described by Hefti and Smith (2000) in the auditory cortex. They proposed that IB cells may be well-suited to generate synchronized bursts of activity that match the frequency of cortical oscillations and it has also been hypothesized that bursting cells may be particularly important for information processing (Lisman, 1997). The possibility that regular spiking (RS) neurons in layer V also contribute to the corticocollicular projection, providing less robust but more specific information about sensory stimuli, has also been put forward (Bajo and Moore, 2005) on the basis of morphological similarities between the neurons projecting selectively to the CNIC in the gerbil and the RS cells described in rat auditory cortex (Hefti and Smith, 2000). Less clear is the role played by layer VI cells that project to the IC due to the morphological variety of the cortical cells involved and the absence of further functional studies.

All auditory cortical fields are thought to participate in the corticocollicular projection (Winer et al., 1998). The primary areas of the auditory cortex most heavily target the IC, whereas non-primary areas project mainly to tegmental areas and the superior colliculus (SC). For example, in the ferret, injections of fluorescent microbeads in the IC (Figures 3A,B) label cells that are located mainly in the middle ectosylvian gyrus (MEG, Figure 3C), where the primary areas, A1, and the anterior auditory field, AAF, are located. In contrast, when tracer injections are placed in the SC, labeled cells are more prominent in the posterior and anterior regions of the gyrus (Bajo et al., 2010b) where secondary and association cortical areas are located (Bizley et al., 2005).

Neurons projecting to the IC are segregated within A1, with different cortical regions targeting different locations in the dorsomedial region of the IC. In rats, Saldaña et al. (1996) have shown that the location of the terminal fields in the dorsal cortex of the IC (DCIC) and the CNIC changes from dorsolateral to ventromedial as the cortical injection sites are placed more anterior in Te1 (corresponding to A1), and according to the well-described tonotopic arrangement in both structures.

The corticocollicular projection is predominantly ipsilateral, but about 15% of the neurons comprising this pathway terminate in the contralateral IC (Bajo et al., 2010a), and these cells originate only in layer V (Schofield, 2009). Although neurons that project ipsi- or contralaterally are intermingled throughout the auditory cortex, only a small proportion project to both sides (Figure 2). After injecting tracers in each IC, Coomes et al. (2005) found a small number of double labeled cells, and these formed a much larger proportion (up to 80%) of the weaker contralateral projection than of the corticollicular neurons on the ipsilateral side (6%) (Figure 2, arrows).

## Segregation of the corticofugal input: do subcortical auditory neurons receive a common input?

Given that the corticofugal pathway links the cerebral cortex with many auditory structures, including the medial geniculate body (MGB), IC, superior olivary complex (SOC), cochlear nuclei (CN), sagulum, and the paralemniscal regions [reviewed in Winer (2007); Malmierca and Ryugo (2010)], an important question is whether individual cortical neurons target more than one subcortical region via long-range collaterals or whether these projections are segregated. This will determine whether descending corticofugal influences selectively affect specific subcortical regions or have a more general influence on subcortical processing.

Although not all combinations have been studied, the evidence currently available [reviewed in Lee et al. (2011)] indicates that less than 10% of corticofugal neurons with long-branching axons innervate multiple nuclei. Regarding the corticocollicular cells, no neurons have been found that target both the IC and the MGB (Wong and Kelly, 1981), but neurons projecting to both the IC and either the caudal striatum (Moriizumi and Hattori, 1991), SOC, or CN (Doucet et al., 2003) have been described.

It therefore appears that the auditory cortex modulates thalamic, midbrain, and brainstem neurons via a set of parallel channels that largely originate from different cortical cells. However, there are technical limitations in studying long-range collaterals. With anterograde tracers, it is difficult to follow individual axons for long distances and visualize terminal fields in regions that are far apart. Injecting different combinations of retrograde tracers into corticofugal target structures can be used to visualize double or triple labeled cells bodies in the cortex (Schofield et al., 2007), but this approach tends to underestimate the size of these projections because the injection sites rarely cover the full extent of the structures in question.

Physiological studies combining stimulation of layer V projection neurons with simultaneous recordings from each IC or from the IC and other brainstem nuclei provide an alternative method for investigating the extent to which these pathways are divergent, but this approach has so far been limited to examining corticofugal inputs to individual target nuclei. In addition to the presence of some collateral projections to multiple targets, it is possible that nearby cortical output cells connected by profuse local axonal branching (Lee et al., 2011), and therefore sharing common properties, may modulate auditory and non-auditory neurons located in different thalamic, midbrain, and brainstem regions.

Schofield (2010) has recently proposed that corticofugal projections from layer V form two general patterns with potentially different functions. In most cases, these projections have single subcortical targets, consistent with descending influences operating via independent, parallel projections. The minority of cortical neurons with divergent projections to multiple targets presumably exert more widespread influences on subcortical processing of auditory signals.

## Terminal fields in the inferior colliculus: different IC subdivisions

Evidence for the existence of a corticocollicular projection was actually provided using anterograde tracing techniques before the cortical projection neurons had themselves been characterized. In fact, the use of the axon degeneration technique led to the first observation of degenerating fibers in the monkey midbrain after lesions had been made in the temporal cortex (Thompson, 1901). Later Diamond et al. (1969) used the degeneration method of Nauta and Gygax to reveal the presence of this projection in cats and established its key features, which are now known to apply across different species. First, all fields of the auditory cortex send fibers to the IC bilaterally, although more sparsely on the contralateral side. Second, the corticocollicular pathway targets the three IC subdivisions, with CNIC receiving the smallest input. Third, corticocollicular fibers in the CNIC are oriented in parallel laminae.

**Figure 4:**
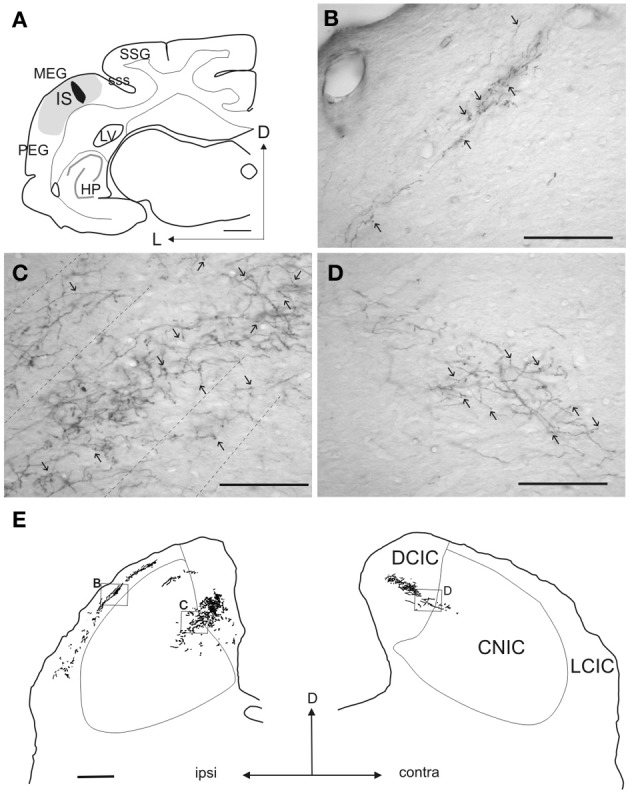
**Terminal fields in the IC after a tracer injection in the ferret auditory cortex. (A)** Coronal section at the level of the left auditory cortex showing the location of a rhodamine injection site in the center of the MEG where A1 is located. The halo of the injection site is shown in gray while the center is in black. **(B–D)** Examples of anterograde terminal fields in the IC at the locations indicated by the boxes in **(E)**. Calibration bars: 1 mm **(A)**, 100 μm **(B–D)**, 0.5 mm **(E)**. Modified with permission from Bajo et al. (2007).

With these methods, however, it was difficult to demonstrate that the degenerated fibers observed actually terminate in the IC itself, but this was subsequently confirmed using different anterograde neural tracers in a large variety of mammals (tamarin, Luethke et al., 1989; squirrel monkey, Fitzpatrick and Imig, 1978; cat, Andersen et al., 1980; Winer et al., 1998; ferret, Bajo et al., 2007; rat, Coleman and Clerici, 1987; Herrera et al., 1994; Saldaña et al., 1996; gerbil, Budinger et al., 2000). An example of cortical terminal fields in the IC is shown in Figure 4. In this case, a large rhodamine injection was made in ferret A1 (Figure 4A), which produced a symmetrical pattern of terminal labeling in each IC. As expected, the ipsilateral IC was most heavily labeled (Figure 4E). Cortical axons mainly innervated the dorsomedial region of the IC, with terminal fields more profuse in the DCIC than in the dorsal part of the CNIC. The labeled axons in the CNIC were also thinner and their terminals smaller, with lower bouton density, than those in the DCIC (Figures 4C,D). In the lateral cortex (LCIC) of this animal, a network of fibers was observed with many terminals and *en passant* boutons (Figure 4B).

The corticocollicular pathway has been described most extensively with anterograde tracers in the cat by Winer et al. (1998), who made tracer injections in each of 12 auditory or auditory-related cortical fields. They defined the corticocollicular pathway as a divergent and convergent system, with individual cortical areas projecting to several IC subdivisions and each IC subdivision receiving convergent inputs from multiple cortical fields. In addition, they stressed the importance of the primary cortical areas in this projection, the fact that corticocollicular axons mainly target IC regions that receive less ascending input, and raised the possibility that at least two parallel cortical systems may exist that target the IC cortices and CNIC independently.

**Figure 5:**
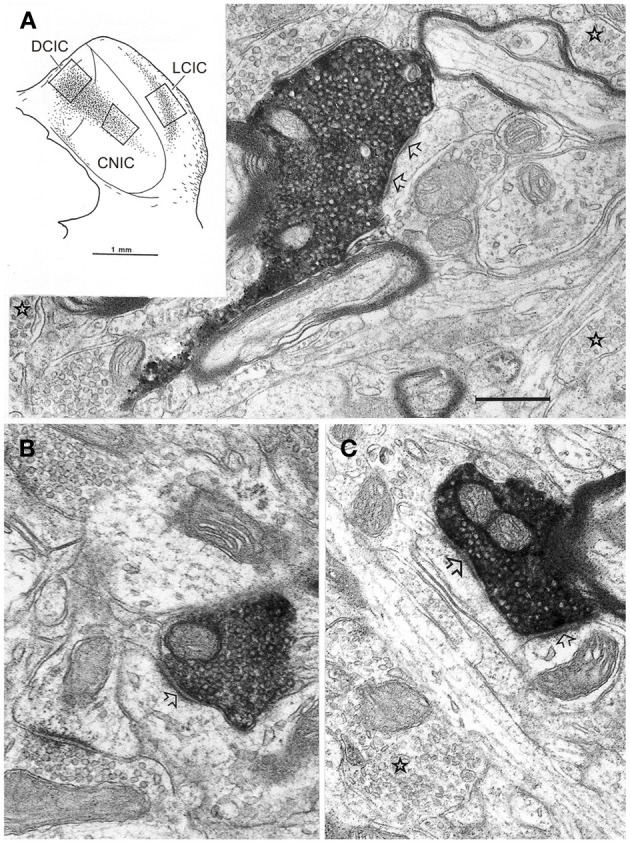
**Electron micrographs of labeled endings in the three main subdivisions of the IC after a large injection of biotinylated dextran amine was made in the ipsilateral primary auditory cortex in the rat.** Labeled terminals in DCIC **(A)**, CNIC **(B)**, and LCIC **(C)** contain round vesicles and make asymmetric synaptic contacts (arrows). Unlabeled terminals with pleomorphic vesicles are also observed (stars). Inset in panel **(A)** shows the distribution of corticocollicular terminal fields and the areas (the box in each IC subdivision) that were used for electron microscopy. Calibration bar: 0.4 μm. Modified with permission from Saldaña et al. (1996).

Only two studies have analyzed the corticollicular terminals at an ultrastructural level. Jones and Rockel (1973) examined degenerated boutons in the IC of cats in which cortical lesions had been made, while Saldaña et al. (1996) labeled terminal boutons in the rat IC after making biotinylated dextran amine injections in A1 (Figure 5). Labeled boutons in every IC subdivision contained round synaptic vesicles and made asymmetric synaptic contacts (Figure 5, arrows), which are generally thought to be features of excitatory synapses (Peters et al., 1991). This is consistent with evidence for the glutamatergic nature of this projection as demonstrated by a decrease in D-aspartate release in the different IC subdivisions following auditory cortex ablation (Feliciano and Potashner, 1995). Corticocollicular fibers synapse mainly on distal dendritic profiles, including dendritic spines, with few contacts on cell bodies or large dendrites (Saldaña et al., 1996).

Although these morphologic features suggest that corticocollicular axons may be non-driving, electrical stimulation of A1 has been found to evoke short latency (1.0–1.4 ms) excitatory responses and longer latency IPSPs in IC neurons (Massopust and Ordy, 1962; Mitani et al., 1983). This is more consistent with a strong driving input from the cortex, although EPSPs with longer latencies can also be evoked, mainly in the CNIC, suggesting a polysynaptic pathway and/or more diffuse influence. Cortical axons have a direct excitatory effect on ascending colliculogeniculate neurons as well as an indirect inhibitory effect via GABAergic collicular interneurons (Mitani et al., 1983).

## Topographic organization of corticocollicular inputs

In view of the physiological studies described in the next section, the corticocollicular projection to the CNIC deserves particular attention. The magnitude of this pathway has been a matter of controversy for several years, and it is possible that species differences may exist. On the other hand, variations in the results reported may be more a consequence of differences in the techniques used in these studies and the difficulty in interpreting negative results in tracing experiments.

**Figure 6:**
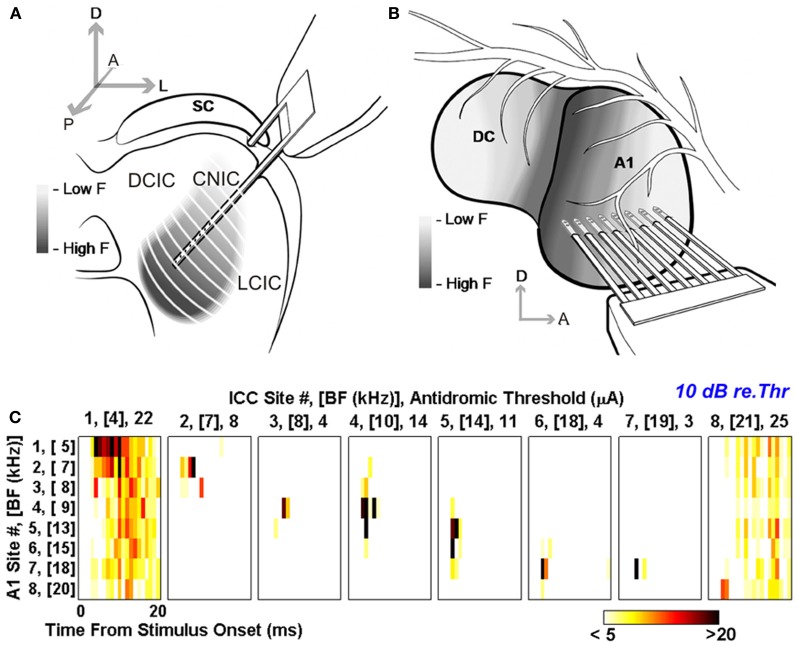
**Antidromic activation reveals tonotopically organized projections from A1 to the CNIC in guinea pig.** Multi-site probes were positioned along the tonotopic axis of the guinea pig CNIC **(A)** and A1 **(B)**. **(C)** Post-stimulus time histograms (PSTHs) for eight A1 locations with different best frequencies (BF) from low (location 1) to high (location 8). These responses were evoked by antidromic stimulation equivalent to 10 dB above threshold at eight frequency-matched locations in the IC. Color scale corresponds to the total number of spikes across 40 trials where any values ≤5 and ≥20 were set to white and black, respectively. Modified with permission from Lim and Anderson (2007).

Anterograde tracing studies have shown that the location of the terminal fields in the CNIC varies topographically with the location of the injection sites in A1 (Saldaña et al., 1996; Bajo et al., 2007). For example, Bajo et al. (2007) found that injecting tracers at two distinct locations in ferret A1, where neurons were tuned to different frequencies, produced two distinct bands of labeling in the CNIC, suggesting that the A1-CNIC projection links neurons in both structures with similar frequency tuning. This has been confirmed physiologically in the guinea pig by positioning multi-site probes along the tonotopic axes of A1 and the CNIC (Lim and Anderson, 2007). After electrically stimulating different locations in the CNIC, these authors recorded antidromic-evoked activity in A1 locations with matching best frequencies (Figure 6). By linking neurons with common sound frequency preferences, this topographic projection provides an anatomical substrate for corticofugal modulation of IC neurons with related receptive field properties.

## Corticofugal modulation of auditory processing in the inferior colliculus

Attempts to investigate the role of descending corticofugal projections in auditory processing have utilized two approaches [reviewed in Suga (2012)]. First, effects on the response properties of subcortical neurons have been examined following focal electrical stimulation of A1. Second, the complementary approach of inactivating the cortex, either locally or globally, has been used in an attempt to remove the influence of descending projections. In most cases, electrophysiological recordings have been carried out to examine whether such manipulations of cortical activity result in transient changes in the response properties of subcortical neurons. Cortical inactivation has also been used to determine the extent to which the effects on subcortical processing of activating other brain areas, such as the nucleus basalis in the basal forebrain, are mediated via cortical circuits.

**Figure 7:**
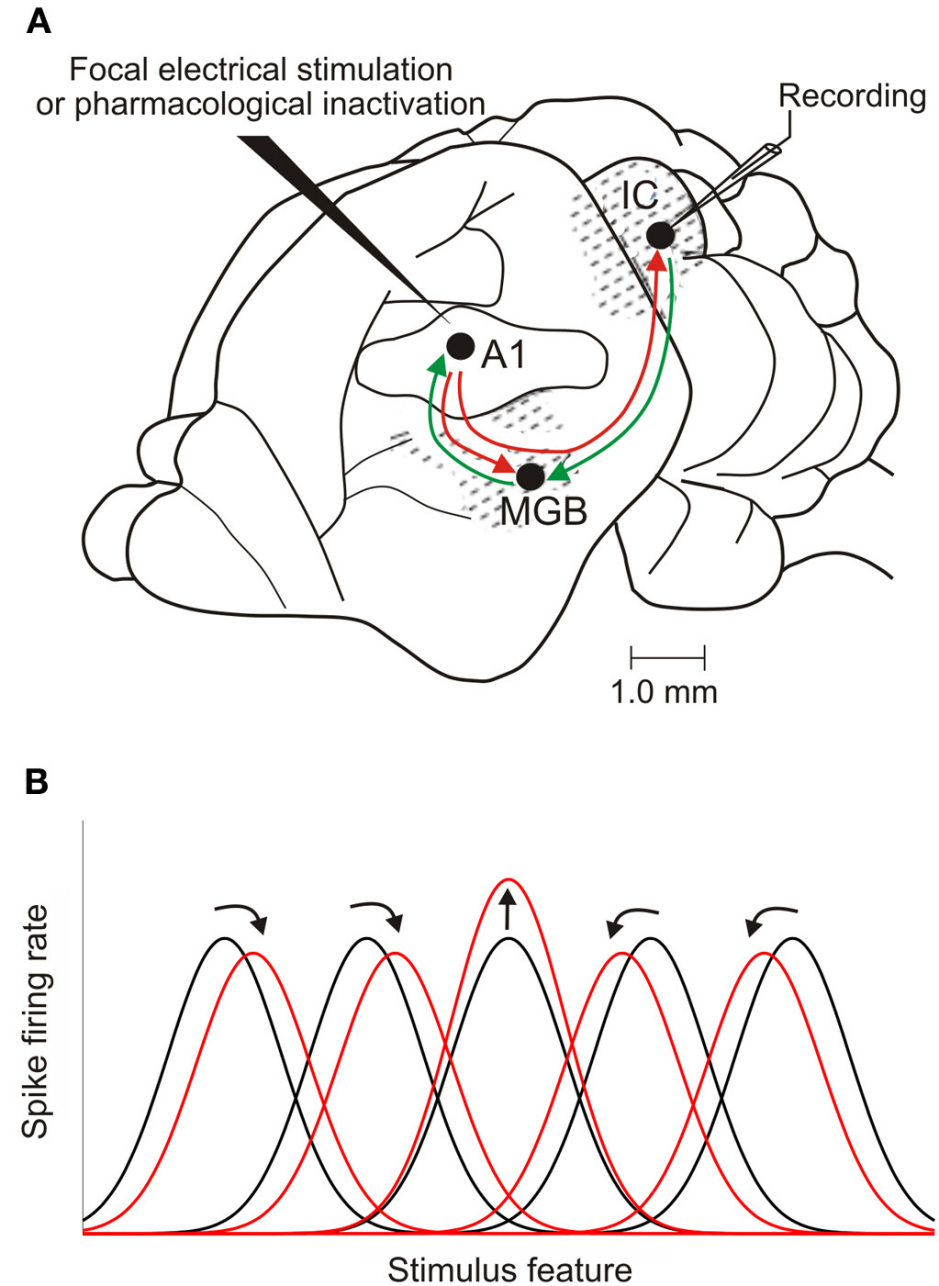
**Corticofugal modulation of IC response properties. (A)** Lateral view of the brain of a mustached bat, one of the species used most in cortical stimulation and inactivation experiments. The auditory cortex has reciprocal ascending and descending connections with the medial geniculate body (MGB) in the thalamus. It also sends a descending projection to the IC, which, in turn, projects to the MGB. **(B)** Focal electrical stimulation in the cortex results in facilitation of the responses of IC neurons that have tuning properties matched to those of cortical neurons at the site of the stimulating electrode. The tuning of unmatched IC neurons may shift toward that of the stimulated cortical neurons (as illustrated here), resulting in an expanded representation of the stimulus feature. Shifts in tuning away from that of the stimulated cortical neurons have also been described, compressing the midbrain representation. Adapted with permission from Suga (2012).

**Figure 8:**
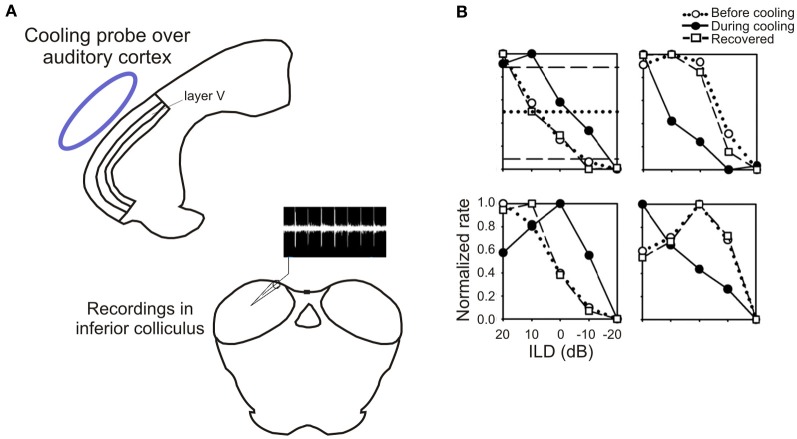
**Inactivation of the auditory cortex alters sensitivity to interaural level differences (ILD) in the inferior colliculus. (A)** Schematic of the experimental setup, showing a cooling probe (blue) above the auditory cortex and a recording electrode in the ipsilateral IC. **(B)** Examples of rate-ILD functions obtained before, during and after cortical inactivation. Adapted with permission from Nakamoto et al. (2008).

These studies have provided considerable evidence that corticofugal inputs make an important contribution to the response properties of neurons at multiple subcortical levels. In the IC, neuronal sensitivity to sound frequency (Ma and Suga, 2001a; Yan et al., 2005), intensity (Yan and Ehret, 2002), duration (Ma and Suga, 2001b), and location (Zhou and Jen, 2005; Nakamoto et al., 2008) changes after manipulating activity in A1 (Figures 7, 8). Thus, the descending system can shape the way midbrain neurons respond to stimulus attributes, such as sound frequency or intensity, which are initially encoded in the cochlea, as well as those that rely on central processing, like sound source location. This suggests that cortical feedback is likely to influence the representation of multiple sound features in the midbrain, implying widespread effects on auditory perception. Recent research also suggests that the auditory cortex modulates collicular processing of simultaneously presented harmonic complexes, suggesting a possible role for descending projections in segregating different sound sources (Nakamoto et al., 2010).

The nature of the changes evoked by focal cortical stimulation or inactivation depends on the similarity between the response properties of the neurons in the cortex and the midbrain (Yan and Suga, 1998; Ma and Suga, 2001b; Yan and Ehret, 2002; Jen and Zhou, 2003; Yan et al., 2005; Zhou and Jen, 2005). For example, if IC neurons are tuned to the same frequency as the cortical neurons recorded at the site of the stimulating electrode, their best frequencies remain the same after the cortex is activated and, in some studies, their frequency tuning is sharpened [reviewed by Suga (2012)]. By contrast, if the neurons are tuned to different frequencies, the best frequencies of the IC neurons are typically shifted toward those of the activated cortical neurons (Figure 7). In a similar vein, the magnitude of the changes induced in the minimum thresholds, dynamic ranges and both the sound duration and azimuth tuning of IC neurons varies with how closely their response properties are matched to those of the neurons activated in the cortex (Suga, 2012).

The dependence of these effects on the relative frequency preferences of the neurons is consistent with the topographic nature of the corticocollicular pathway. Modulation of IC response properties appears to involve a combination of cortically evoked facilitation of the responses of IC neurons that have corresponding tuning properties and inhibition in unmatched neurons. Consequently, these descending inputs provide a route by which localized increases in cortical activity can selectively enhance auditory processing in specific regions of the IC. This, in turn, will presumably provide stronger input via the thalamus to that part of cortex, further modulating the representation of potentially important stimuli at higher levels of the auditory system.

Most studies have focused on the effects of electrically stimulating or inactivating neurons in A1, although Yan and Suga (1996) also examined the influence of descending inputs originating in the frequency modulation-frequency modulation (FM-FM or FF) cortical area of the mustached bat on the sensitivity of IC neurons to biosonar pulse-echo combinations. Similarly, although all subdivisions of the IC receive descending cortical inputs, the recording studies appear to have been restricted mainly to the CNIC. Given that corticocollicular inputs primarily target other regions of the IC, it seems likely that the changes recorded in the response properties of CNIC neurons are mediated, at least in part, via polysynaptic pathways involving the DCIC or LCIC.

## Corticofugal modulation and auditory plasticity

The changes induced in the response properties of IC neurons following local manipulation of cortical activity have been reported to last for periods ranging from a few seconds to several hours (e.g., Zhang and Suga, 2000; Zhou and Jen, 2005). Corticofugal projections may therefore contribute to the selective processing of sounds that acquire behavioral significance as a result of learning. For example, combining tones of a particular frequency with a mild electric shock causes the best frequencies of A1 neurons to undergo a relatively long-lasting shift toward that value (Bakin and Weinberger, 1990; Weinberger et al., 1993). Training-induced improvements in sound discrimination are also accompanied by plasticity in the response properties of A1 neurons (e.g., Polley et al., 2006; Schnupp et al., 2006). Whether perceptual learning results in comparable changes at subcortical levels as a result of corticofugal feedback is unknown, but the experience-dependent plasticity produced by auditory fear conditioning is seen not only in the cortex, but also subcortically in both the thalamus (Edeline and Weinberger, 1991) and IC (Gao and Suga, 1998). This shift in best frequencies in the IC closely resembles that evoked by cortical electrical stimulation and is abolished by global inactivation of A1, indicating that it is mediated by corticocollicular feedback (Gao and Suga, 1998).

An involvement of the corticofugal system in representational plasticity in the auditory system has also been demonstrated by studies in which behavioral salience is simulated by electrical stimulation of the nucleus basalis, the region of the cholinergic basal forebrain that projects to the neocortex (Casamenti et al., 1986; Rasmusson et al., 1992). The resulting release of acetylcholine facilitates auditory thalamocortical synaptic transmission and increases cortical excitability (Metherate and Ashe, 1993). Stimulus-specific changes in cortical receptive fields are produced by pairing electrical stimulation of the nucleus basalis with sound presentation, which are very similar to those seen after behavioral training (Bakin and Weinberger, 1996; Kilgard and Merzenich, 1998; Yan and Zhang, 2005). Thus, neuronal best frequencies are shifted toward those of the paired stimuli. This pairing paradigm also induces shifts in the best frequencies of IC neurons, which are dependent on activity in the auditory cortex (Zhang et al., 2005), providing further evidence for the role of corticofugal descending connections in experience-dependent plasticity.

Although studies utilizing either electrical stimulation or inactivation of A1 have provided valuable insights into the influence of corticofugal projections on the response properties of neurons in other brain regions, these methods are not particularly selective. Thus, related changes in the auditory responses and tuning properties of neurons have been reported at multiple levels of the auditory pathway, including both the thalamus and cochlear nucleus, as well as neighboring regions of the cortex [reviewed by Suga (2012)]. The extent to which the changes observed in the IC following manipulation of A1 activity represent direct effects of corticocollicular modulation or the indirect involvement of other areas that provide inputs to the IC remains unclear. More importantly, none of the studies considered so far has examined the behavioral consequences of activating or deactivating the corticocollicular projection, and so the function of this descending pathway in auditory processing remains unclear.

**Figure 9:**
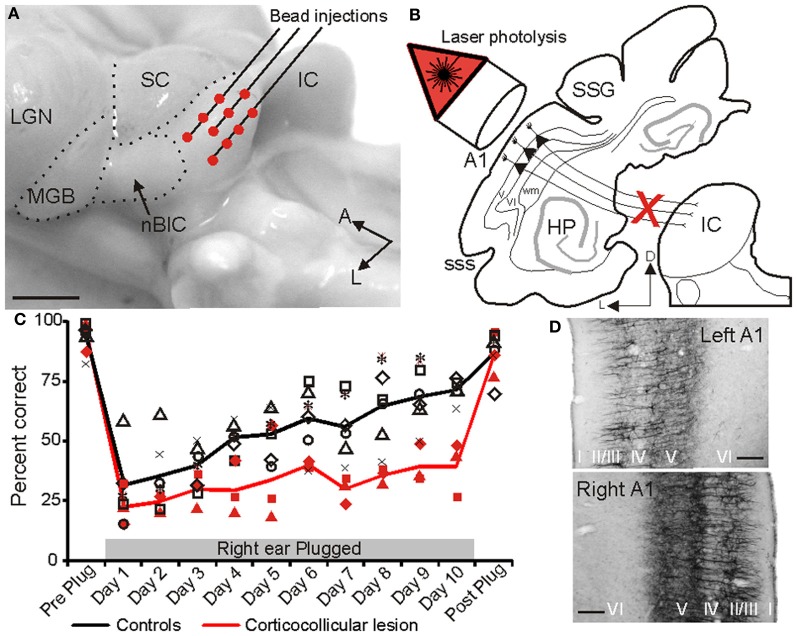
**The projection from the auditory cortex to the inferior colliculus is essential for training-induced plasticity of spatial hearing in adult ferrets. (A)** Lateral view of the ferret midbrain showing the location and number of injections of fluorescent microspheres conjugated with chlorine e_6_ in the left IC. **(B)** Schematic showing the selective ablation of retrogradely labeled layer V corticocollicular neurons by illumination of the ipsilateral auditory cortex with near-infrared light. **(C)** Sound localization accuracy (averaged across 12 speaker locations in the horizontal plane) before the right ear was plugged (Preplug), on each of the 10 days over which an ear plug was worn and following its removal (Post-plug). Data from control animals are shown in black and from the ferrets with corticocollicular lesions in red. The symbols represent different animals and the lines show the mean scores. **(D)** Staining with the SMI_32_ antibody, a marker of layer III and layer V pyramidal cortical neurons, was sparser in the left (lesioned) primary auditory cortex, resulting in a less distinct bilaminar appearance (top) than on the right side (bottom). Calibration bars: 2 mm in **(A)** and 0.1 mm in **(D)**. Modified with permission from Bajo et al. (2010a).

To address this question, Bajo et al. (2010a) used a chromophore-targeted neuronal degeneration technique to investigate the behavioral effects in ferrets of selectively eliminating layer V pyramidal cells in the primary auditory cortical areas that project to the IC (Figure 9). This involved retrogradely labeling corticocollicular projection neurons by injecting fluorescent microbeads conjugated with chlorine e_6_ in the IC on one side of the brain, and subsequently illuminating the ipsilateral auditory cortex with near-infrared light. This resulted in a loss of about two thirds of the A1 neurons that project to the IC, without affecting those in surrounding cortical areas. As previously discussed, most corticocollicular axons target the ipsilateral IC, so this approach allowed an assessment of the effects of removing descending axons predominantly on one side of the brain only.

Given that cortical electrical stimulation (Zhou and Jen, 2005) or inactivation (Nakamoto et al., 2008; Figure 8) alters the spatial response properties of IC neurons, Bajo et al. (2010a) examined the effects of eliminating corticocollicular neurons on auditory localization and its recalibration by experience. Although they observed no change in sound localization accuracy, the training-induced plasticity that normally occurs in adult ferrets after disrupting the available spatial cues by occluding one ear was severely impaired (Figure 9C). Thus, while control animals recover their ability to localize sounds accurately despite the continued presence of a plug in one ear, this was not the case in ferrets in which the corticocollicular projection had been largely removed, suggesting that descending pathways are essential for recalibration of the brain's representation of auditory space. This learning deficit was most pronounced in the hemifield contralateral to the lesioned pathway, implying that corticofugal modulation of each IC mediates plasticity in the opposite hemifield.

What information the auditory cortex provides to IC neurons via these descending projections to allow auditory spatial learning to take place is presently unknown. One mechanism for adapting to the presence of an earplug in one ear would be to adjust the sensitivity of auditory neurons to binaural localization cues. Recent studies have shown that the sensitivity of IC neurons to interaural level differences (ILDs; Dahmen et al., 2010) and interaural time differences (ITDs; Maier et al., 2012) can change following short-term adaptation to stimulus statistics, and, as previously illustrated (Figure 8), cortical cooling can produce pronounced alterations in collicular firing-rate-ILD functions. Behavioral evidence, however, suggests that adaptation to a conductive hearing loss in one ear takes place by learning to reweight different localization cues in favor of the monaural spectral localization cues provided by the non-occluded ear rather than by remapping the altered binaural cues (Kacelnik et al., 2006; Kumpik et al., 2010). It has been shown that individual IC neurons can carry information about ILDs, ITDs, and spectral cues using different neural coding strategies (Chase and Young, 2008), suggesting that corticofugal modulation of specific aspects of their spike discharge patterns could change the contribution of each cue to the output of the neurons and therefore to the perception of sound source location.

The corticocollicular projection is, however, unlikely to work is isolation in mediating the experience-dependent plasticity of spatial coding. Thus, behavioral adaptation by adult ferrets to a unilateral earplug is also impaired following midline lesions of the olivocochlear bundle (Irving et al., 2011), which originates in the superior olivary complex where sensitivity to binaural cues is first derived. This suggests that activation of olivocochlear neurons, which are in turn innervated by the IC (Vetter et al., 1993), could produce a frequency-specific adjustment in cochlear output, potentially altering the localization cue sensitivity of neurons at higher levels of the auditory pathway. These findings again highlight the influence of descending pathways at multiple levels of the auditory pathway and the difficulty in isolating the functions of specific projections.

## Concluding remarks

It is becoming increasingly clear that the descending projections found throughout the auditory pathway can have a considerable impact on stimulus processing. This has been demonstrated most clearly for the projection from the auditory cortex to the IC, which is now known to be part of the circuitry responsible for the plasticity and learning observed in the adult brain. Thus, experience-dependent changes in the receptive field properties of cortical neurons that result from classical conditioning or behavioral training can, in turn, trigger a reorganization of subcortical processing. Furthermore, there is growing evidence from studies in which auditory brainstem responses to complex sounds have been recorded in humans that language (Krishnan et al., 2005), musical experience (Kraus and Chandrasekaran, 2010), and auditory training (Carcagno and Plack, 2011; Song et al., 2012) can all modify subcortical processing. This is again thought to reflect the corticofugal modulation of neural processing at subcortical levels, and the IC in particular, where the relevant stimulus features are represented most precisely. Consequently, the plasticity in neuronal response properties that underlies a change in perceptual skills may actually occur subcortically, which, in turn, will alter the information delivered to the cortex.

Although electrical stimulation and inactivation studies have shown that the auditory cortex can facilitate or inhibit the responses of IC neurons according to how well-matched their response properties are, we still know relatively little about how corticofugal modulatory effects are mediated or the relationship between these signals and other inputs to the IC. Because of its pivotal position within the auditory pathway as a site of convergence of bottom up signals from multiple brainstem areas, descending inputs to the IC are likely to play a particularly important role in auditory processing. Nevertheless, the corticocollicular projection is only one of several descending pathways in the auditory system. If we are to understand the role of these circuits, it will be necessary to make greater use of cutting edge techniques for imaging and selectively manipulating the activity of specific cell types and their projections during behavior.

### Conflict of interest statement

The authors declare that the research was conducted in the absence of any commercial or financial relationships that could be construed as a potential conflict of interest.

## Abbreviations

I, II, III, VI, V, VI: cortical layers I–VI
A: anterior
A1: primary auditory cortex
AEG: anterior ectosylvian gyrus
AVCN: anteroventral cochlear nucleus
CN: cochlear nucleus
CNIC: central nucleus of the inferior colliculus
Contra: contralateral
D: dorsal
DC: dorsocaudal field in the guinea pig auditory cortex
DCIC: dorsal cortex of the IC
DCN: dorsal cochlear nucleus
DNLL: dorsal nucleus of the lateral lemniscus
HP: hippocampus
IC: inferior colliculus
ILD: interaural level difference
Ipsi: ipsilateral
IS: injection site
L: lateral
LCIC: lateral cortex of the inferior colliculus
LGN: lateral geniculate nucleus
LSO: lateral superior olive
LV: lateral ventricle
M: medial
MEG: middle ectosylvian gyrus
MGB: medial geniculate body
MSO: medial superior olive
nBIC: nucleus of the brachium of the IC
P: posterior
PEG: posterior ectosylvian gyrus
ps: pial surface
pss: pseudosylvian sulcus
s: section
SC: superior colliculus
SOC: superior olivary complex
SSG: suprasylvian gyrus
sss: suprasylvian sulcus
VNLL: ventral nucleus of the lateral lemniscus
wm: white matter.
